# The role of SH3GL3 in myeloma cell migration/invasion, stemness and chemo-resistance

**DOI:** 10.18632/oncotarget.12231

**Published:** 2016-09-24

**Authors:** Ruoying Chen, Hong Zhao, Dan Wu, Chen Zhao, Weiling Zhao, Xiaobo Zhou

**Affiliations:** ^1^ Department of Radiology, Wake Forest School of Medicine, Medical Center Boulevard, Winston-Salem, NC, USA; ^2^ Department of Blood Transfusion, The Second Affiliated Hospital of Harbin Medical University, Harbin, Heilongjiang Province, China; ^3^ College of Computer Science and Software Engineering, Shenzhen University, Shenzhen, China

**Keywords:** CD138^−^ cells, SH3GL3, migration/invasion, stemness, chemo-resistance

## Abstract

Multiple myeloma (MM) is an incurable cancer characterized by clonal expansion of malignant plasma cells in the bone marrow and their egress into peripheral blood. The mechanisms of myeloma cells migration/invasion have remained unclear. Herein, we found SH3GL3 was highly expressed in the CD138-negative (CD138^−^) myeloma cells. The migration/invasion capability of CD138^−^ cells was significantly higher than that in the CD138-positive (CD138^+^) cells. Silencing SH3GL3 using shRNA reduced myeloma cells migration/invasion. Conversely, overexpression of SH3GL3 increased myeloma cells migration/invasion. Moreover, SH3GL3 is also associated with the stemness and chemo-resistance of CD138^−^ myeloma cells. Elevated expression of stem cell and multi-drug resistant markers were seen in the myeloma cells with overexpressed SH3GL3; while knocking-down SH3GL3 reduced the expression of these markers. A marked increase in *p*-PI3K and *p*-FAK was observed in the cells with overexpressed SH3GL3. To test if FAK/PI3K signaling pathway was involved in the SH3GL3-mediated myeloma cells migration, the cells transfected w/wo SH3GL3 cDNA were treated with FAK inhibitor 14 and PI3K inhibitor LY294002. Inhibition of FAK and PI3K attenuated SH3GL3-mediated migration /invasion. Our findings indicate that SH3GL3 plays an important role in myeloma cell migration/invasion, stemness and chemo-resistance. The SH3GL3-mediated myeloma cell migration/invasion is mediated by FAK/PI3K signaling pathway.

## INTRODUCTION

Multiple myeloma (MM) is the second most prevalent hematologic malignancy. It is characterized by the clonal expansion of malignant plasma cells [1] and remains largely incurable in spite of the advent of several therapeutic strategies [2]. The clinical outcomes of MM patients are extremely heterogeneous, with survival ranging from several months to more than 15 years [3]. Even if MM patients may reach a complete remission initially with currently available chemo- and radio-therapy, most MM patients eventually developed relapsed disease after several years. A number of studies have suggested that a small fraction of cells responsible for disease relapse is capable of clonogenic growth and resistant to the therapeutic drugs [4, 5]. This subpopulation is termed as multiple myeloma stem cells [4, 5]. Recent studies indicated that clonogenic multiple myeloma cells lacked the expression of plasma cell marker CD138 [6] were enriched in CD138^−^ CD19^−^ CD38^++^ plasma cells, while CD19^+^ B cells never formed MM colonies [7]. In contrast, Matsui et al. [4] found that CD138^−^ CD19^+^ CD27^+^ cells isolated from the peripheral blood of MM patients were able to engraft in the NOD/SCID mice and generate mature CD138^+^ myeloma cells.

Myeloma cell migration is very important for myeloma cell invasion and dissemination. However, the mechanism regulating cell migration remains unclear. Myeloma cells are thought to enter blood vessels in the periphery and then migrate to the bone marrow (BM) microenvironment by extravasting from the vascular endothelium. The interaction between myeloma cells and BM stromal cells promotes myeloma cell migration to secondary sites in the BM [8]. Studies have found that circulating malignant plasma cells were present in more than 70% of MM patients [4, 9]. A number of studies have shown that several proteins participate in the migration of myeloma cells, such as insulin-like growth factor-1 (IGF-1) [10, 11], stromal cell-derived factor-1α [12], wingless-ints (Wnt) [8], and integrin β-7 [13]. However, the complexity of migration process makes it likely that other molecules may also play critical roles.

In order to characterize the differences between CD138-positive and CD138-negtive cells, Yang et al. [14] performed gene expression profiling analysis and identified that a group of genes were upregulated in CD138^−^ subgroup, including mammalian SH3-domain GRB2-like 3 (SH3GL3). The mammalian SH3GL3 is also known as endophilin A3 and extra eleven nineteen (EEN)-B2, and belongs to a small family of SH3 domain containing proteins [15]. Endophilins contain a Bin/Amphiphysin/Rvs (BAR) domain, a variable region and an SH3 domain [15]. The BAR domain protein superfamily are important for cellular traffic. SH3 domains play critical roles in protein-protein interaction and are involved in signal transduction processes, such as the regulation of cancer cell migration/invasion and adhesion [16, 17]. Endophilins also play important roles in receptor tyrosine kinase signaling, mitochondrial network dynamics, and synaptic vesicles retrieval [15].

Previous studies have shown that SH3GL3/endophilin A3 is associated with the adaptor protein Cb1-interacting protein of 85K (CIN 85). Ubiquitin ligase Cb1 recruits endophilin-CIN85 complex to mediate the internalization of receptor tyrosine kinases [18]. SH3GL3 is preferentially expressed in mammalian brain and testes, and can interact with Huntingtin exon 1 protein, dynamin, and synaptojanin [19]. Delic et al. [20] found that SH3GL3 was involved in glioma cell invasion and silence of SH3GL3 resulted in a reduction in the cumulative activity of matrix metalloproteins in these cells. To date, the specific function of SH3GL3 in cancer has yet to be addressed.

SH3GL3-associated signaling pathways are still poorly understood. A few of studies have focused on the role of endophilins in endocytosis. Aramaki et al. found that SH3GL3/endophilin A3 interacted directly with metastasis-associated protein 1 [21]. Endophilin-3-interacting protein 1 contains numerous SH3-domain binding sites, and it functions as an endocytic protein that affects energy homeostasis via its interaction with endophilins [22]. Endophilin A1 can be phosphorylated by Rho-associated protein kinase (ROCK) and leads to a reduction in EGFR endocytosis [23]. Phosphorylation of Endophilin A2 by a FAK/Src complex inhibited endophilin/dynamin interactions and abrogates endocytosis of MT1-MMP [17]. The interaction of Ataxin 2 with endophilin A1/A3, adaptor protein CIN85, and ubiquitin ligase c-CbI regulates endocytic receptor cycling [24]. The role of SH3GL3 in MM has not been investigated.

In this study, we found that CD138^−^ myeloma stem-like cells had a higher migration and invasion capability. SH3GL3 was highly expressed in CD138^−^ myeloma cells. Knocking-down SH3GL3 using shRNA reduced myeloma cell migration and invasion and decreased the stemness and chemo-resistance. In contrast, overexpression of SH3GL3 increased myeloma cell migration/invasion and enhanced the stemness and chemo-resistance of myeloma stem cells. Activation of FAK and PI3K were required for SH3GL3-increased myeloma cell migration/invasion. Inhibition of FAK or PI3K attenuated SH3GL3-enhanced cell migration. Taken together, these findings reveal, at the first time, that the SH3GL3-induced cell migration/invasion is mediated by FAK/PI3K signaling pathway.

## RESULTS

### Characterization of CD138^−^ isolated from U266 myeloma cells

**Figure 1:**
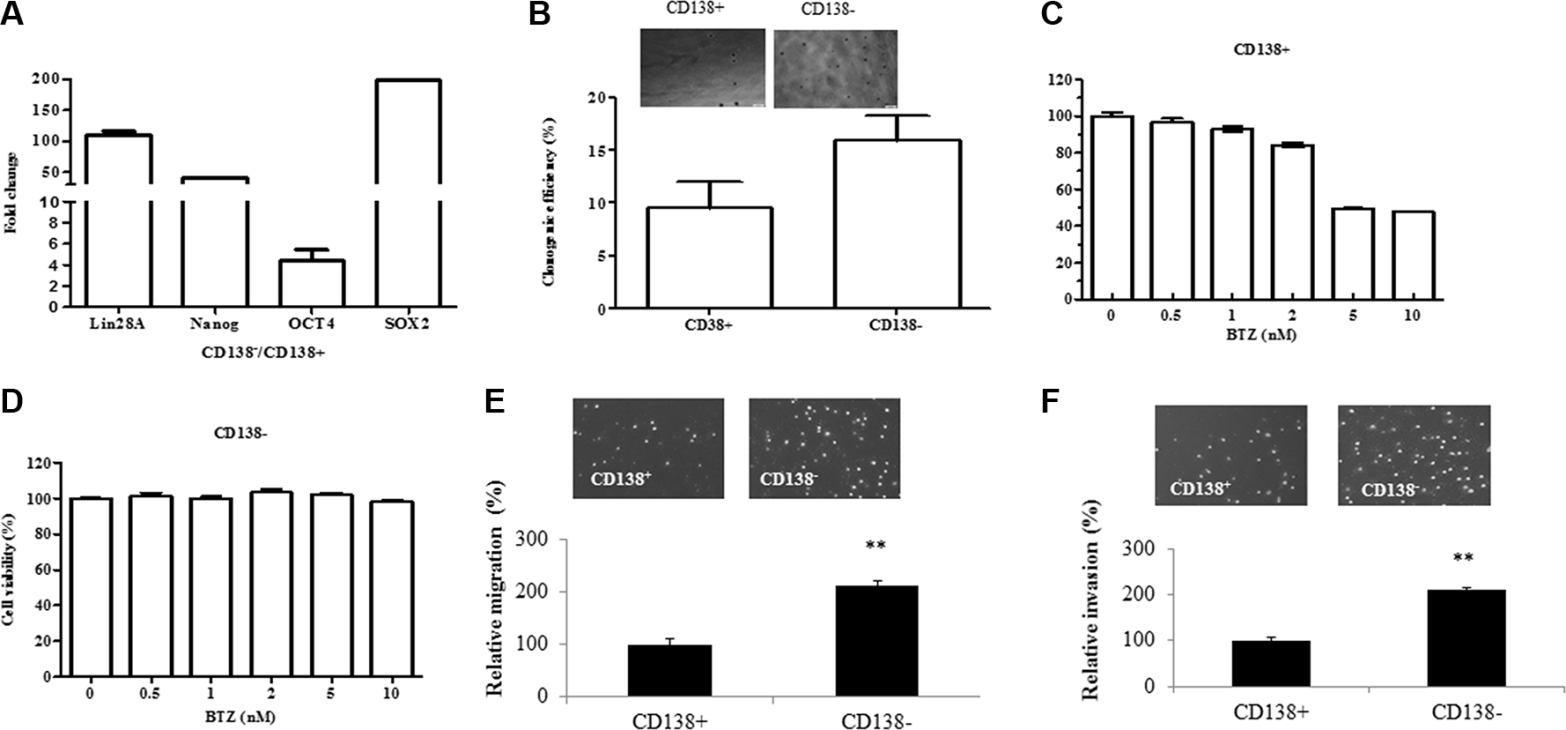
CD138^−^ cells display the characteristics of stem cells and have greater migration and invasion capability (**A**) The expression of stem cell markers including Lin28A, Nanog, OCT4 and Sox2 was examined in the CD138^+^ and CD138^−^ U266 cells using qRT-PCR. (**B**) CD138^+^ and CD138^−^ U266 cells display different clonogenic capability. (**C**–**D**) MTT assay showed the responses of CD138^+^ and CD138^−^ U266 cells to the treatment with various concentration of BTZ. The relative value (%) was calculated as the ratio of the number of treated cells and the number of untreated cells. (**E**) Transwell migration assay demonstrated the migration of CD138^−^ and CD138^+^ cells. (**F**) Transwell matrigel-coated invasion assay showed the invasion of CD138^−^ and CD138^+^ cells. The relative value (%) was calculated as the ratio of the number of CD138^−^ and the number of CD138^+^. The results are representative of 3 independent experiments and shown as mean ± SE, **p < 0.01.

CD138^+^ and CD138^−^ cells were isolated from U266 myeloma cells using autoMACS and CD138 microbeads. CD138^−^ cells account about 3–4% of the total number of cells. Oct4, Lin28A, Nanog and Sox-2 are pluripotency-associated transcription factors that maintain the self-renewal and pluripotency of embryonic *stem* cells. We measured the expression of Lin28A, Nanog, OCT4, and Sox2 in the CD138^+^ and CD138^−^ cells using qRT-PCR. As shown in the Figure 1A, these genes were highly expressed in the CD138^−^ U266 myeloma cells, when compared with that in the CD138^+^ cells. The expressions of stem markers were also determined in other three myeloma cell lines (Supplementary Figure S2). It is known that clone formation ability reflects a self-renewal capacity, which is a characteristic of tumor stem cells. To examine the clonogenic capacity of both subpopulations, we carried out the soft agar clonogenic assay. As shown in the Figure 1B, CD138^−^ cells had a higher clonogenic capacity than that in the CD138^+^ subpopulation. CD138^−^ cells from other three cell lines also displayed higher clonogenic capacity (Supplementary Figure S3). We also measured the sensitivity of both subpopulations to the therapeutic drug Bortezomib (BTZ). The CD138^+^ and CD138^−^ cells were treated with 0–10 μM BTZ for 72 hours, and cell viability was determined using MTT assay. As shown in the Figure 1C, the treatment CD138^+^ cells with 2, 5 and 10 μM BTZ led to 16%, 51% and 54% reduction in cell viability, respectively, when compared to non-treated controls. In contrast, CD138^−^ cells were resistant to BTZ treatment. Incubating CD138^−^ cells with 0–10 μM BTZ for 72 h failed to affect cell viability (Figure 1D). Taken together, these results have demonstrated that CD138^−^ cells have the properties of stem cells and are resistant to BTZ treatment.

### CD138^−^ cells exhibit a higher migration/invasion capability

To evaluate the migration/invasion capacity of CD138^−^ and CD138^+^ cells, we measured cell migration and invasion using transwell assay. Comparing with the CD138^+^ cells, we observed a more than two-fold increase in the number of CD138^−^ cells migrated into the lower chamber (Figure 1E). Cell invasion was measured by assessing the migration of cells through matrigel-coated transwell filters overnight. Similarly, the number of CD138^−^ cells invaded through matrigel was more than twice as much as the CD138^+^ cells (Figure 1F). Our data indicates that CD138^−^ cells have a higher migration and invasion capability.

### Overexpression of SH3GL3 enhances migration and invasion of myeloma cells

**Figure 2:**
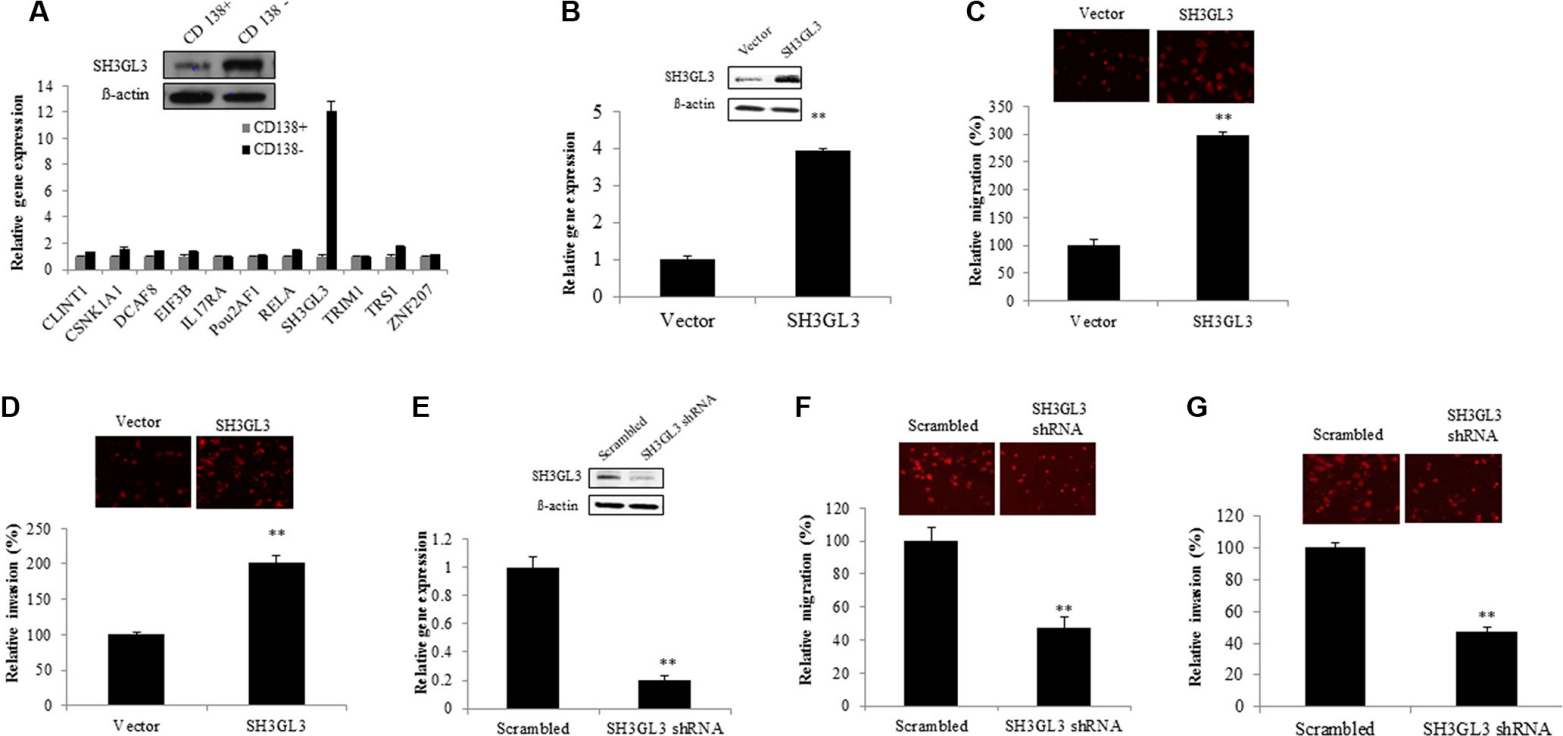
Overexpression of SH3GL3 enhances the migration and invasion of H929 myeloma cells; while knocking-down SH3GL3 leads to a significant reduction in U266 myeloma cells migration/invasion (**A**) The expression of selected genes in the CD138^+^ and CD138^−^ U266 cells was determined using qRT-RCR (lower panel). SH3GL3 was highly expressed in CD138^−^ U266 cells and this gene was verified using western blotting at the protein level (upper panel). (**B**) The protein (upper panel) and mRNA (lower panel) levels of SH3GL3 in H929 cells infected with SH3GL3 cDNA lentiviral particles (expressed as SH3GL3) and empty vectors. (**C**) Transwell migration assay showed the number of migrated cells. (**D**) Transwell matrigel-coated invasion showed the number of invaded cells. (**E**) Western blotting and qRT-PCR analysis showed the protein (upper panel) and mRNA (lower panel) levels of SH3GL3 in the U266 cells infected with SH3GL3 shRNA lentiviral particles and scrambled vector. (**F**) Transwell migration assay showed the number of migrated cells. (**G**) Transwell matrigel-coated invasion showed the number of invaded cells. The relative value (%) was calculated as the ratio of the number of treated cells and the number of vector control. Cells were labeled using Dil dye. The images were acquired with an Olympus microscope. The results are representative of 3 independent experiments and shown as mean ± SE, **p < 0.01.

The microarray analysis from Yang et al. [14] suggests that CD138^+^ and CD138^−^ cells have distinct gene expression profiles. We measured the mRNA levels of several genes in CD138^+^ and CD138^−^ U266 cells using qRT-PCR. We found that SH3GL3 was highly expressed in the CD138^−^ cells, and verified the protein level using western blotting in CD138+ and CD138-cells as shown in the Figure 2A. To test if SH3GL3 plays a role in myeloma cell migration and invasion, we first overexpressed SH3GL3 in a myeloma cell line. Human H929 myeloma cells expressed a relative low level of SH3GL3. We overexpressed SH3GL3 in this cell line. H929 cells were infected with lentiviral particles containing cDNA construct or empty vector. The mRNA and protein levels of SH3GL3 were determined using qRT-PCR and western blotting, respectively. A 3.8 fold increase of SH3GL3 mRNA level was seen in the SH3GL3 cDNA-infected cells, compared with the cells infected with empty vector (Figure 2B, bottom). The protein level of SH3GL3 was also increased in the SH3GL3 cDNA-infected cells (Figure 2B, top). H929 cell infected with SH3GL3 cDNA or empty vector were plated into the transwell inserts and the cells invaded into the bottom of the filter were counted. Overexpression of SH3GL3 resulted in a 3-fold increase in cell migration (Figure 2C). The number of cells invaded through the bottom of the matrigel membrane were also significantly elevated (Figure 2D). Our data indicated that overexpression of SH3GL3 enhanced migration and invasion of myeloma cells.

### Knocking-down SH3GL3 leads to a significant reduction in cell migration and invasion

To further confirm the role of SH3GL3 in regulating myeloma cell migration and invasion, we knocked down the expression of SH3GL3 using SH3GL3 shRNA in U266 cells. U266 myeloma cells were infected with SH3GL3 shRNA lentiviral particles. As shown in the Figure 2E, infection of cells with SH3GL3 shRNA resulted in a ~80% reduction in SH3GL3 expression, determined using qRT-PCR (Figure 2E, bottom). The protein level of SH3GL3 was also markedly reduced (Figure 2E, top). As shown in the Figure 2F, knocking-down SH3GL3 led to a significant reduction in the number of migrated cells, when compared with the cells infected with scrambled shRNA lentiviral particles. The number of invading cells was also reduced significantly (Figure 2G). Thus, knocking-down SH3GL3 appears to play a critcal role in decreasing myeloma cell migration and invasion.

### Molecular mechanisms regulating MM cells migration induced by SH3GL3

**Figure 3:**
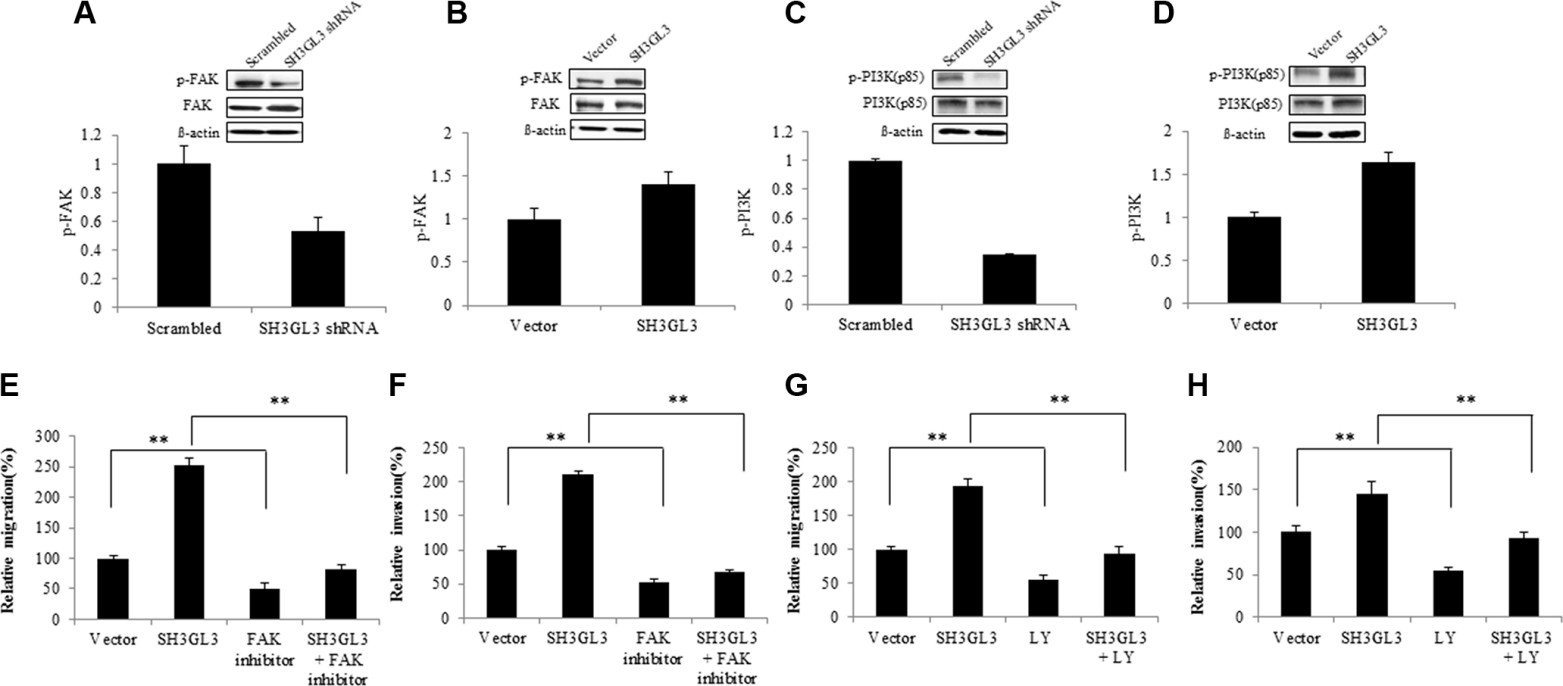
SH3GL3-induced myeloma cells migration/invasion is mediated through the activation of FAK/PI3K signaling pathway (**A**–**B**) Western blotting analysis showed the protein level of FAK and *p*-FAk in the cells infected with SH3GL3 shRNA (A) or cDNA (B) lentiviral particles. (**C**–**D**) Wester blotting analysis showed the protein level of PI3K (85kd) and *p*-PI3K (85kd) in the cells infected with SH3GL3 shRNA (C) or cDNA (D) lentiviral particles. (**E**–**F**) H929 cells infected with SH3GL3 cDNA lentiviral particles were pretreated with FAK inhibitor 14 (10 μM) for 1 hour and then cells were loaded into the insert in 300 μL of serum-free RPMI 1640 medium. After 24 hours incubation at 37°C, the cells in the lower chamber were counted. The relative migration was shown (E) and the relative invasion was shown (F). (**G**–**H**) H929 cells infected with SH3GL3 cDNA lentiviral particles were pretreated with PI3K inhibitor LY294002 (50 μM) for 1 hour and then cells were loaded into the insert in 300 μL of serum-free RPMI 1640 medium. After 24 hours incubation at 37°C, the cells in the lower chamber were counted. The relative migration was shown (G) and the relative invasion was shown (H). The relative value was calculated as the ratio of the number of treated cells and the number of vector control. The results are representative of 3 independent experiments and shown as mean ± SE, **p < 0.01.

We then sought to determine the signaling pathways involving in the SH3GL3-activated migration of myeloma cells. Previous studies have reported the signaling pathways involved in the regulation of myeloma cell migration [8, 10–13]. The focal adhesion kinase p125 (FAK) is a cytoplasmic tyrosine kinase and plays an important role in cell migration [25]. Phosphatidylinositol-3 kinases (PI3Ks) are a large family of lipid enzymes that can phosphorylate the 3′-OH group of phosphatidylinositol on the plasma membrane [26]. A study has demonstrated that the PI3K signaling pathway is involved in many different cellular processes such as motility, metabolism, cell survival and cell progression [27]. Recent results have indicated that the FAK and PI3K were involved in the migration of myeloma cells [10]. We, therefore, assessed the status of FAK activation in myeloma cells with overexpressed or silenced SH3GL3. As shown in Figure 3A, knocking-down SH3GL3 led to a significant reduction in the phosphorylated FAK protein level. In contrast, overexpression of SH3GL3 remarkably enhanced the phosphorylation of FAK (Figure 3B). We also analyzed the phosphorylated protein level of p85-PI3K. A similar pattern of changes in the phosphorylated protein levels of p85-PI3K was observed in the myeloma cells with reduced- or over- expression of SH3GL3 (Figure 3C and 3D).

Our data has shown that SH3GL3 regulated the activation status of FAK and p85-PI3K. Here, we investigated if the SH3GL3-activated cell migration and invasion were regulated through FAK/PI3K signaling. The cells infected with SH3GL3 shRNA or cDNA lentiviral particles were treated with either 10 μM FAK inhibitor (FAK inhibitor 14) or 50 μM PI3K inhibitor LY294002 for 1 h prior to seeding onto the transwell inserts. Figure 3E and 3F show that preincubation of cells with FAK inhibitor 14 abolished the SH3GL3-promoted cell migration and invasion. Inhibition of PI3K using LY294002 also abrogated SH3GL3-induced migration and invasion in myeloma cells (Figure 3G and 3H). These results indicated that FAK and PI3K were key mediators in the SH3GL3- activated myeloma cell migration and invasion.

**Figure 4:**
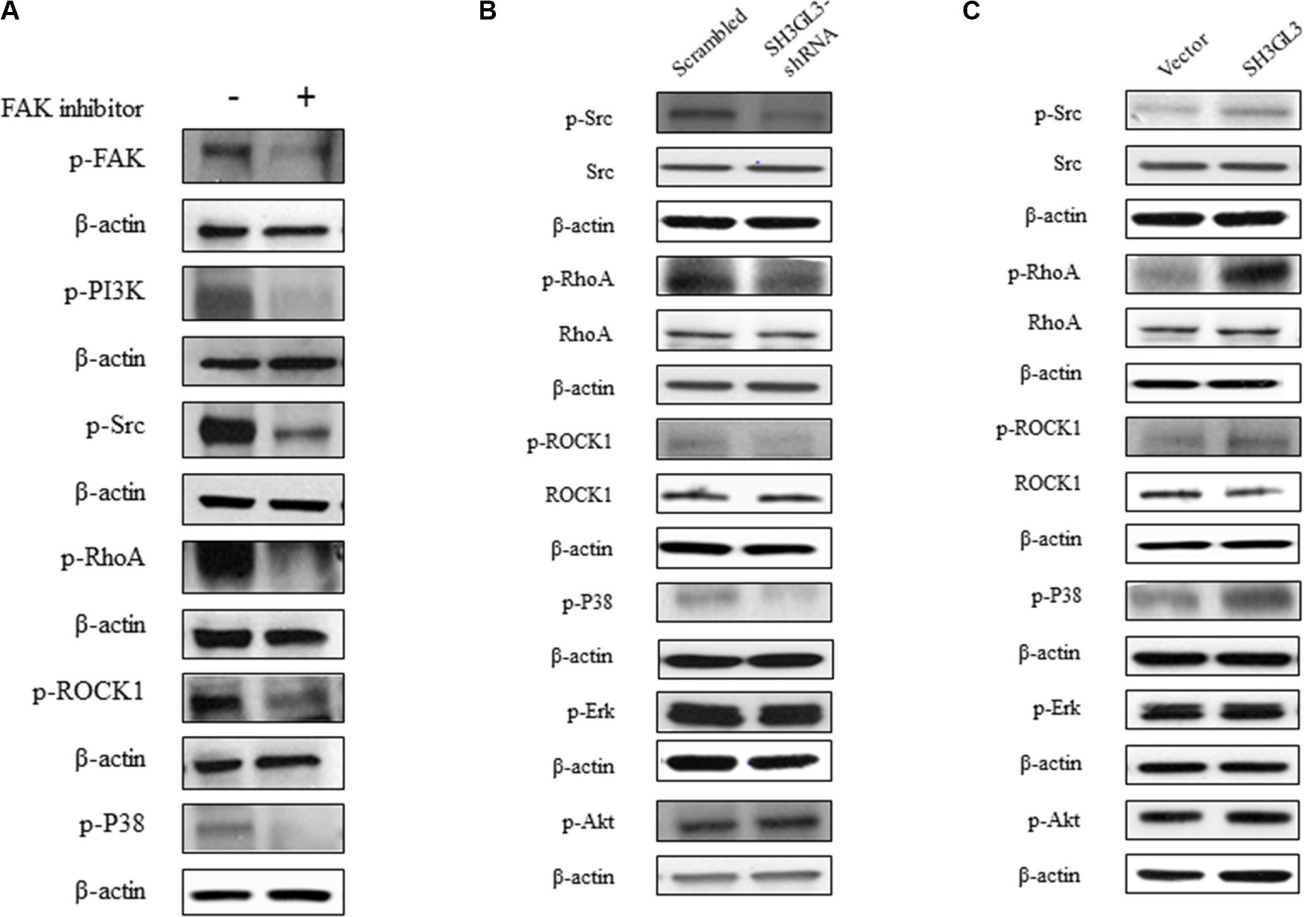
The activation status of Src, RhoA, ROCK1, P38, ERK, and AKT in the myeloma cells treated with FAK inhibitor or myeloma cells with suppressed and overexpressed SH3GL3 (**A**) The phosphorylated protein levels of FAK, PI3K, Src, RhoA, ROCK1, and P38 in the U266 cells treated with FAK inhibitor were decreased. (**B**) Representative blots showed the total and phosphorylated protein levels of Src, RhoA, ROCK1, P38, ERK and AKT in the U266 cells infected with SH3GL3 shRNA. (**C**) Representative blots showed the total and phosphorylated protein levels of Src, RhoA, ROCK1, P38, ERK and AKT in the U266 cells infected with SH3GL3 shRNA in the H929 cells infected with SH3GL3 cDNA. β-actin is loading control.

To determine the effect of FAK on the downstream signaling proteins, U266 cells were treated with FAK inhibitor and the related proteins were analyzed using western blotting. As shown in the Figure 4A, inhibition of FAK resulted reduction in the protein levels of *p*-PI3K, *p*-Src, *p*-RhoA, *p*-ROCK1and *p*-P38. We didn't see any changes on other signaling molecules, such as ERK and AKT (data not shown). Our data indicated that PI3K, Src, RhoA, ROCK1 and P38 are involved in the FAK signaling. To determine the effect of SH3GL3 on FAK signaling pathway, the total and phosphorylated protein levels in the cells with infected shRNA or cDNA of SH3GL3 were compared with the non-infected counterparts. As shown in the Figure 4B, knocking-down SH3GL3 led to a reduction in the protein levels of *p*-Src, *p*-RhoA, *p*-P38 and *p*-ROCK1. Conversely, overexpression of SH3GL3 enhanced the activation of these proteins (Figure 4C).

**Figure 5:**
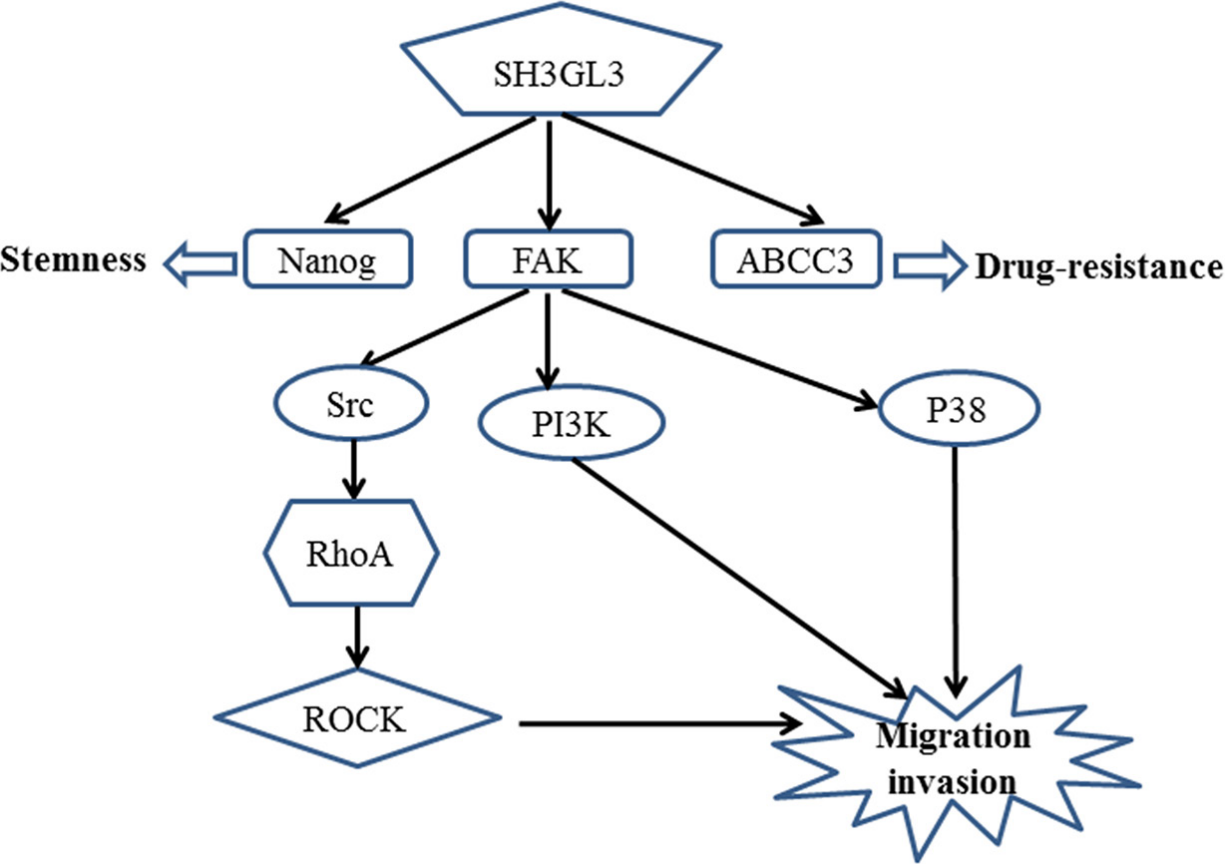
A schematic representation of the proposed mechanism for the SH3GL3-mediated migration/invasion, stemness and drug resistance of myeloma cells

Based on the results that we obtained above and the published signaling pathways for FAK, we proposed a potential mechanism associated with the SH3GL3-activated migration and invasion in the myeloma cells as shown in the Figure 5.

### Overexpression of SH3GL3 promotes myeloma cell stemness and enhances the expression of drug-resistant proteins

**Figure 6:**
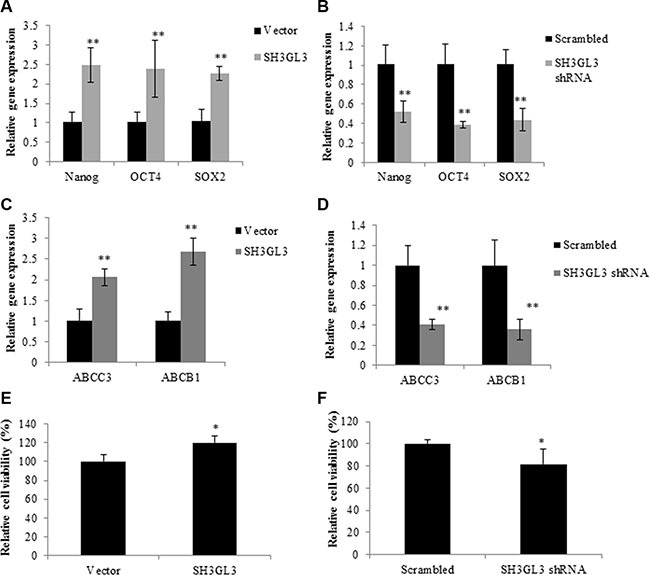
SH3GL3 regulates the expression of stem cell markers and drug resistance genes Gene expression of Nanog, OCT4 and SOX2 was determined in the H929 cells infected with SH3GL3 cDNA (**A**) or U266 cells infected with SH3GL3 shRNA (**B**). qRT-PCR analysis showed the expression of the multi-drug resistance genes, ABCC3 and ABCB1, in the H929 cells infected with SH3GL3 cDNA (**C**) or U266 with SH3GL3 shRNA (**D**). The cells infected with SH3GL3 cDNA (**E**) or shRNA (**F**) were treated with BTZ (10 nM) and cell viability was determined using MTT assay. The relative value was calculated as the ratio of treated cells and vector control. All experiments have been repeated 3 times, Mean ± SE, **p < 0.01, *p < 0.05.

To test whether SH3GL3 was indeed the driver rather than an associated phenomenon of stem cell features and drug resistance, we measured the expression of stem cell markers (Nanog, OCT4, Sox2 and Lin28A) in the H929 cells infected with SH3GL3 cDNA and empty vector using qRT-PCR. Overexpression of SH3GL3 led to a marked increase in the expression of Nanog, OCT4 and Sox2, respectively (Figure 6A). In contrast, knocking-down SH3GL3 using shRNA suppressed the expression of these stem cell markers (Figure 6B). No changes were observed in the expression of Lin28A in the cells with either overexpressed or silenced SH3GL3, when compared with their corresponding controls (data not shown). We also investigated the involvement of SH3GL3 in drug resistance. We measured the expression of multi-drug resistant markers, including multi-drug resistant protein 3 (ABCC3) and ATP-Binding Cassette Sub-Family B Member 1 (ABCB1). As shown in the Figure 6C and 6D, the expression of ABCC3 and ABCB1 were remarkably increased in the cells with overexpressed SH3GL3 (Figure 6C), and significantly reduced in the SH3GL3-silenced U266 cells (Figure 6D). We subsequently examined the response of cells to BTZ. Myeloma cells infected with lentiviral particles containing SH3GL3 cDNA, shRNA or vectors were incubated with 5 nM BTZ for 72 h and cell viability was determined using MTT assay. Interestingly, overexpression of SH3GL3 resulted in an increase in cell viability when compared with the cells infected with empty vectors (Figure 6E). In contrast, the cells infected with SH3GL3 shRNA showed a reduction in cell viability compared with the scrambled control (Figure 6F). We also determined the role of SH3GL3 in cell response to melphalan and lenalidomide. U266 cells infected with SH3GL3 or scrambled shRNA were treated with 5 μM melphalan or 10 μM lenalidomide for 72 h, respectively. Knocking-down SH3GL3 sensitized the U266 cells to melphalan, but not lenalidomide (Supplementary Figure S4). Taken together, these results suggest that SH3GL3 plays a potential role in the stemness and drug resistance of myeloma cells to BTZ and melphalan.

## DISCUSSION

Most tumors are hierarchically organized and sustained by a subset of clonogenic cells that have self-renewal potentials. These clonogenic cells can grow clonally into tumors and have potential to metastasize to other sites. Disease relapse indicates that the cells responsible for tumor regrowth are relatively drug resistant. Recent studies indicate that multiple myeloma contains heterogeneous cell types with different clonogenic potential [6]. The clonogenic myeloma cells exhibit properties of stem cells and are resistant to drug treatment [4, 28]. CD138 is expressed in malignant and normal plasma cells, but not on virgin/ naive B cells, memory B cells, T cells, or monocytes [29]. Previous studies have shown that human myeloma cell lines and clinical specimens consist of only a minority of cells that lacked CD138 expression [6]. These CD138^−^ cells display more clonogenic potential *in vitro* and *in vivo* than CD138^+^ plasma cells and exhibit stem cell properties [4, 30]. In the past several years, many efforts have been focused on clonogenic myeloma cells and their roles in myeloma initiation and relapse [6, 14]. However, the exact mechanism and their functional roles in the disease process are yet to be elucidated.

Though much progress in investigating clonogenic myeloma progenitor cells has been made, it is still debated whether CD138^−^ cells are myeloma stem cells. In our study, we isolated CD138^−^ cells from myeloma cell line U266, NCI H929, RPMI8226, and MM1.S using autoMACS separator and CD138 microbeads, and measured the expression of stem cell markers and their clonogenic ability. We found that the CD138^−^ cells isolated from U266 cell line expressed a high level of Lin28A, Nanog, OCT4, and Sox2, and had a higher clonogenic potential compared with the CD138^+^ cells. The CD138^−^ cells isolated from other myeloma cell lines had also higher clonogenic potential. CD138^−^ U266 myeloma cells were resistant to the BTZ. Furthermore, it has been reported that CD138^−^ cells could initiate tumor development and differentiate into CD138^+^ cells *in vivo* [31]. Moreover, CD138^−^ cells from a murine model of MM are quiescent and most invasive [32]. In our study, we also found that CD138^−^ cells exhibited a stronger invasion capability than CD138^+^ cells *in vitro*. Previous studies and our data suggest that CD138^−^ clonogenic myeloma cells are myeloma stem cells with high migration potential.

We have demonstrated the distinct expression of SH3GL3 in CD138^−^ and CD138^+^ U266 myeloma cells consistent with the data from microarray analysis [14]. Paino et al. [33] reported that CD138^++^ and CD138^low^ have similar gene expression and genomic profiles. There are two differences between their study and ours. First, the isolation method they used was different from the one we used. Fluorescence-activated cell sorting was used by Paino et al., and autoMACS separator plus CD138 microbeads was used in our laboratory. Second, they analyzed CD138++ and CD138^low^, but we analyzed CD138^+^ and CD138^−^. Because CD138^−^ cells only account for a small subpopulation, it is more difficult to obtain enough CD138^−^ cells from MM patients. Therefore, gene profiling analysis using microarray has not shown a differential expression of SH3GL3 in the human MM specimen compared to the health control [34]. Delic et al. reported that SH3GL3 regulated glioma cell invasion, and knocking-down SH3GL3 reduced the activity of matrix metalloproteinase [20]. Iwakaki et al. [16] found that SH3 domains were critical to protein-protein interaction and regulated signal transduction process, such as cancer cell invasion. In our study, we found that increased numbers of cells migrated into the lower chamber when SH3GL3 was overexpressed, while few cells in the lower chamber when SH3GL3 was silenced. Our data suggest that SH3GL3 plays an important role in migration and invasion of myeloma cells.

Most of myeloma therapies take advantage of the key features of malignant myeloma cells, such as fast proliferation rate and sensitivity to certain therapeutic drugs, or disrupting signaling pathways and microenvironments required for the survival of terminally differentiated myeloma cells. The combined chemotherapies have led to clinical improvement of MM patients. However, these therapies cannot successfully eradicate all of the cancer cells in most of the patients, because of the existence of myeloma stem cells. In this study, we have demonstrated that SH3GL3 enhanced the stemness of myeloma cells. Overexpression of SH3GL3 in the myeloma cells induced the specific characteristics of stem cells, including the enhanced expression of prototypical stem cell genes and drug resistant genes. These cells were also resistant to BTZ treatment. The opposite effect was observed while SH3GL3 was silenced. These findings indicate that SH3GL3 appears to be an important player in maintaining myeloma stem cell features.

Cell migration plays crucial roles in cancer progression [35]. Focal adhesion kinase (FAK) is a non-receptor kinase that plays critical roles in a variety of biological processes [36]. In the recent years, numerous studies have explored the role of FAK in various cancers [37–39]. FAK^−/−^ embryonic fibroblasts exhibited profound defects in migration *in vivo*, suggesting the role of FAK in promoting cell migration [40]. In the present study, we found that the activation of FAK was stimulated by SH3GL3. Several FAK downstream signaling pathways have been known to involve in FAK-activated cell migration. Previous studies indicated that the FAK/PI3K signaling pathway plays important roles in the regulation of cell migration [41, 42]. Reiske et al. found that PI3K inhibitor (LY294002) inhibited FAK-promoted migration in Chinese hamster ovary (CHO) cells [41]. Our data showed that FAK inhibitor was able to suppress the activity of PI3K. Moreover, inhibition of FAK or PI3K reduced SH3GL3-activated cell migration and invasion. FAK can also increase cell migration through other pathways. Phosphorylation of FAK at Y397 creates a binding site for the SH2 domain of Src and triggers the complex formation of FAK-Src [43]. The FAK-Src complex can control cell shape, focal contact turnover and generation of traction forces events [44, 45]. Our results showed that FAK inhibition led to a significant inhibition of phosphorylated Src in the myeloma cells. It was reported that FAK overexpression could promote the p190RhoGEF (a ubiquitously expressed RhoA-specific GEF) tyrosine phosphorylation in Neuro-2a cells, which is associated with the enhanced activity of p21 RhoA. This event contributes to the regulation of cell migration [46, 47]. ROCK, a downstream effector of RhoA, is also involved in cell migration [48]. RhoA/ROCK signaling pathway plays a crucial role in FAK activation. Therefore, we investigated the RhoA and ROCK as the downstream pathway of FAK. In our studies, the results showed that treating cells with FAK inhibitor reduced the protein levels of phosphorylated RhoA and ROCK, suggesting that they are the downstream signaling molecules of FAK. Additionally, we measured the activation of P38, Akt, and Erk. The activation of P38 was regulated by SH3GL3. Akt is the downstream signaling molecules of PI3K, so we also tested the status of Akt. No significant changes in the *p*-Akt was observed. Although Akt is viewed as a major downstream effector of PI3K, at least in physiological processes, several previous studies suggest that PI3K and Akt act independently of each other in cancers [49–52]. Recent studies have reported that Erk plays crucial roles in cell migration [53, 54]. This protein was also detected in our study, and no significant changes in the *p*-Erk was observed. Our results showed that Akt and Erk were not involved in the SH3GL3-activiated cell migration. Based on our findings, we proposed a mechanism for the SH3GL3-promoted migration and invasion in myeloma cells as shown in the Figure 5. Our study indicates that SH3GL3-activated myeloma cell migration/invasion is regulated through the activation of FAK signaling pathways. Our current *in vitro* findings cannot be simply extrapolated to *in vivo* conditions. Therefore, *in vitro* observations need to be further confirmed using appropriate animal models to support the potential importance of SH3GL3 and related signaling molecules.

It is very important to target clonogenic myeloma cells, which are thought to be responsible for disease process and maintenance of MM [6]. Therapies against terminally differentiated plasma cells may lead to immediate clinical effects. The resistant stem cells may escape from the treatments. Therefore, selectively targeting myeloma stem cells could be prematurely abandoned. In summary, our findings provide the evidence of molecular mechanisms underlying the migration/invasion of myeloma stem cells and offer potential insights into pathways that can be selectively targeted for the treatment of malignant MM disease. SH3GL3 may represent an attractive target for future research.

## MATERIALS AND METHODS

### Cell culture

The human myeloma cell lines U266 and H929 were purchased from the American Tissue Type Culture Collection (Manassas, VA), and cultured in RPMI 1640 medium (Thermo Scientific, Logan, UT) containing 10% heat-inactivated fetal calf serum, 2 mM L-glutamine, 100 U/mL penicillin, and 100 μg/mL streptomycin at 37°C in humidified 5% CO_2_.

### CD138^+^ and CD138^−^ cell isolation

CD138^+^ and CD138^−^ myeloma cells were isolated using autoMACS separator and CD138 microbeads (Miltenyi Biotec Inc., Auburn, CA) according to the manufacturer's instruction. Briefly, 2 × 10^7^ cells were incubated with 40 μL of CD138 microbeads for 15 min at 4°C and resuspended with 500 μL incubation buffer. The cells were then transferred into a separation column, which was placed in a magnetic field in the autoMACS separator. The cells passing through the column were collected as CD138^−^ portion. The magnetically labeled CD138^+^ cells were retained within the column and flushed out using 500 μL incubation buffers. The purity of isolated CD138^−^ cells was more than 94% (Supplementary Figure S1).

### Clonogenic formation assay

Colony formation was performed as described in [14]. Briefly, 5,000 myeloma cells were suspended in 0.35% agarose in 2× complete RPMI medium. The suspensions were placed onto a solidified base layer with 0.5% agarose in 2× complete medium in a 6-well plate. The cells were cultured for three weeks and fresh medium was added to the culture plate every 3–4 days. All plates were imaged and colony numbers counted using Image J software. Only colonies containing more than 50 cells were scored.

### Lentiviral vector construction for SH3GL3 shRNA

To determine the role of SH3GL3 in regulating myeloma cell migration and invasion, SH3GL3 shRNA and scramble sequences were designed using Invitrogen online shRNA software (http://www.sirnawizard.com/siRNA.php, Supplementary Table S1) and cloned into the pLVTHM vector. The constructs were then co-transfected with psPAX2 and PMD_2_G into 293T packing cells using Lipofectamine 2000 (Invitrogen Life Technologies, Carlsbad, CA). After 48 hours, the packaged virus was collected and stored at −80°C until use. The SH3GL3-expressing lentiviral particles were obtained from GeneCopoeia (Rockville, MD). Myeloma cells were infected with SH3GL3 shRNA or SH3GL3-expressing particles in the presence of 8 μg/mL polybrene. The efficiency of SH3GL3 silence and overexpression in the myeloma cells were monitored by qRT-PCR and western blotting. The sequence 1 of shRNA got good results, and this shRNA was used in following assays.

### Real-time RT-PCR (qRT-PCR)

Total RNA was extracted from myeloma cells using an RNeasy Mini kit (Qiagen, Valencia, CA) according to the manufacturer's instructions. First-strand cDNA was synthesized using the SuperScript III RT kit (Invitrogen, Carlsbad, CA). Quantitative real time PCR (qPCR) reactions were carried out on the 7500 Fast Real-time PCR system (Applied Biosystems, Foster City, CA). Briefly, qPCR amplification was performed with 100 ng of cDNA in a 20 μL reaction mixture containing 10 μL 2× All-in-OneTM qPCR mix (GeneCopoeia, Rockville, MD), 300 nM of upstream and downstream primers and nuclear-free water. PCR reaction was conducted with 1 cycle at 95°C for 10 min, 40 cycles at 95°C for 15 s and 60°C for 1 min, followed by dissociation curve analysis. The average ΔΔCT and standard deviation were determined from three independent experiments. The expression of a gene was normalized with the expression of β-actin.

### Western blotting

Myeloma cells were lysed with 1 X RIPA buffer supplemented with protease and phosphatase inhibitor cocktail (Roche Applied Science, Indianapolis, IN) and stored in aliquots at −20°C until use. Twenty micrograms of cell lysates were mixed with an equal volume of Laemmli sample buffer, denatured by boiling, and separated by SDS-PAGE. The separated proteins were then transferred to a nitrocellulose membrane (BioRad, Hercules, CA). The membranes were blocked using 5% non-fat dry milk for 1 h at room temperature and probed with antibodies overnight. After incubated with IgG horseradish peroxidase conjugated secondary antibodies (Cell signaling, Beverly, MA) for 2 h at room temperature, the immunoblots were developed using the enhanced chemiluminescence (ECL) reagent (Cell signaling, Beverly, MA) and visualized using a FluroChemQ processor (Proteinsimple, Santa Clara, CA).

### Cell migration and invasion assay using transwells

For migration studies, the transwell inserts with 8 μm pores in a 24-wells format (Coring Costar, Cambridge, MA) were used. One million cells were fluorescently labeled by incubation with 5 μL of dialkyl carbocyanine membrane dye (Dil; Invitrogen) for 20 min at 37°C. After removing the unbound dyes, 1 × 10^5^ cells in 300 μL of serum-free RPMI 1640 medium were seeded into the upper chamber and 500 μL of RPMI 1640 medium with 100 ng/mL SDF-1 in the lower chamber. After 24 h incubation at 37°C, the cells that migrated into the lower chamber were counted. Triplicates of each experiment were performed.

For the invasion assay, the transwell inserts were coated with 100 μL Matrigel (50 μg/ml) for 4 hours. Dil-labeled myeloma cells (1 × 10^5^) in 300 μL of serum-free medium were loaded into the upper chamber and 500 μL of RPMI 1640 medium with 100 ng/mL SDF-1 in the lower chamber. After incubation for 24–48 hours at 37°C, the cells in the lower chamber was collected and counted.

For the experiments with specific inhibitors, myeloma cells were pretreated with the inhibitors for 1 hour and then loaded into the inserts. The cells in the control groups were treated with equal amount of vehicles. All images were acquired with an Olympus microscope.

### Cell viability assay

Cell viability was determined using a modified thiazolyl blue tetrazolium bromide (MTT) (Acros Organics, Thermo Fisher Scientific, New Jersey) method as described previously [55]. Briefly, 5000 cells /well were plated in 96-well plates and incubated overnight. The cells were treated w/wo bortezomib (various concentration 0.5~10 nM), melphalan (5 μM) and lenalidomide (10 μM) for 72 hours. Then 12.5 μL of 5 mg/mL MTT reagent in PBS was added to each well and incubated for 4 hours in a CO_2_ incubator. Cells were then lysed by adding 50 μL lysis buffer (20% SDS, 50% N, N, N-dimethyl formamide (DMF), PH4.7) at 37°C. The absorbance at 560 nm was measured using a spectrophotometer (Molecular Devices, Sunnyvale, CA).

### Statistical analysis

Results are reported as the mean ± SD for experimental groups performed in three replicate samples. Student *t* test was applied to analyze the statistical significance of the differences between experimental groups. The *P* values less than 0.05 were considered significant.

## SUPPLEMENTARY MATERIALS TABLE FIGURES


