# Supplementary material for: The role of SH3GL3 in myeloma cell migration/invasion, stemness and chemo-resistance
Source: Oncotarget. 2016 Sep 24;7(45):73101–13. doi: 10.18632/oncotarget.12231 (PMC5341966; doi:10.18632/oncotarget.12231)
Supplement: Supplementary file 1 [file oncotarget-07-73101-s001.pdf]

The role of SH3GL3 in myeloma cell migration/invasion, stemness and chemo-resistance

Supplementary Materials

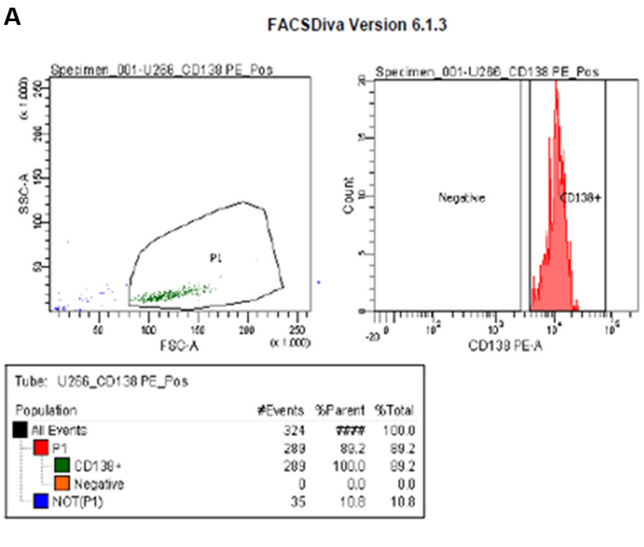

Supplementary Figure S1: The purity of U266 CD138$^+$ and CD138$^-$ after CD138 microbeads. (**A**) The purity of U266 CD138$^+$ is 100%. (**B**) The purity of U266 CD138$^-$ is 94.1%.

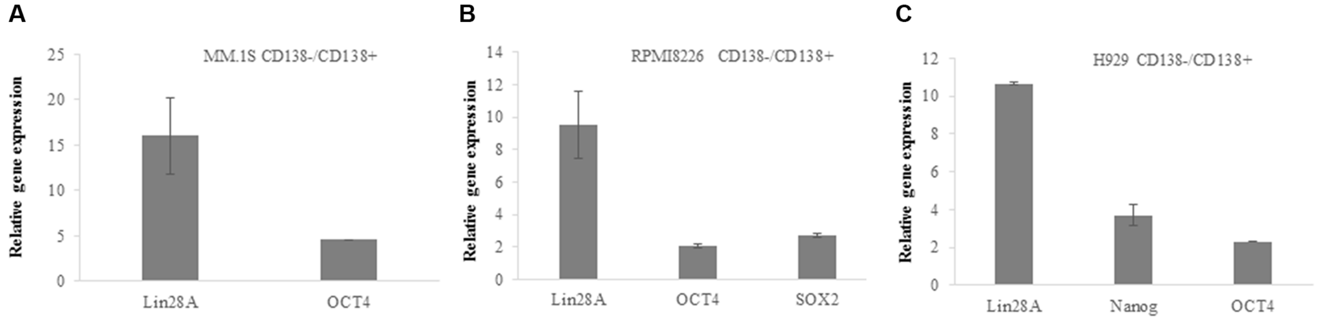

Supplementary Figure S2: The expression of stem cell markers in other myeloma cell lines. The expression of stem cell markers including Lin28A, Nanog, OCT4 and Sox2 was examined in the CD138$^+$ and CD138$^-$ MM.1S (**A**), RPMI8226 (**B**) and H929 (**C**) cell lines derived using qRT-PCR. The relative expression value was calculated as the ratio of CD138$^-$ and CD138$^+$.

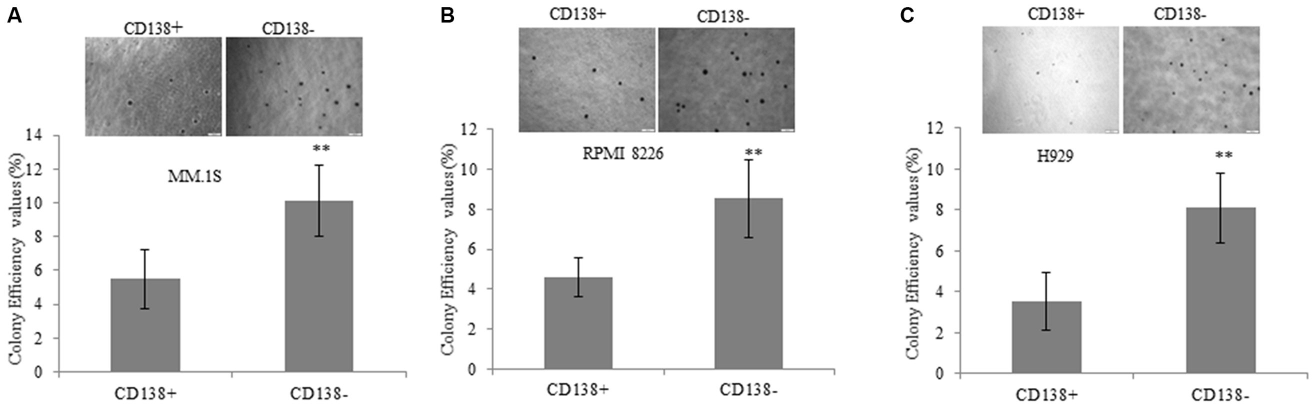

Supplementary Figure S3: Colony efficiency of other myeloma cell lines. $CD138^+$ and $CD138^-$ from MM.1S (**A**), RPMI8226 (**B**) and H929 (**C**) cell lines display different clonogenic capability. $CD138^-$ cells can form more colonies than $CD138^+$.

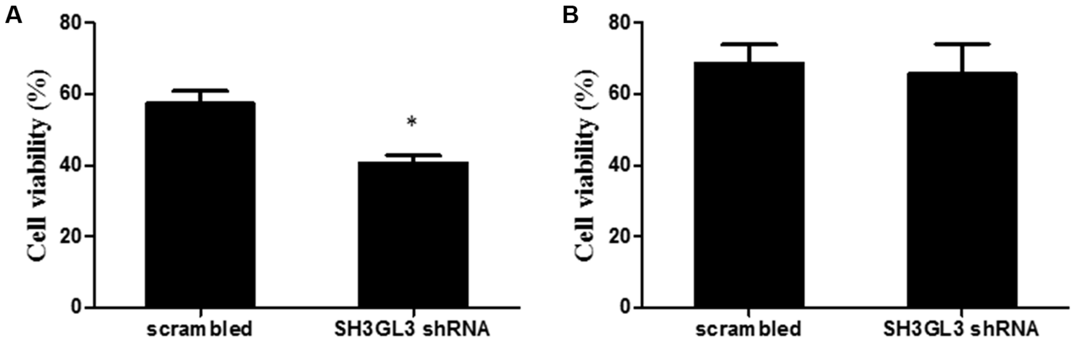

Supplementary Figure S4: Suppresion of SH3GL3 sensitized myeloma cells to melphalan, but not lenalidomide. U266 cells infected with SH3GL3 shRNA or scrambled shRNA were treated with 5 μM melphalan (**A**) or 10 μM lenalidomide (**B**) for 72 hours. The cell viability was determined using MTT assay. All experiments have been repeated 3 times, Mean ± SE, *$p < 0.05$.

Supplementary Table S1: SH3GL3 shRNA sequences designed using siRNA design tool

Seq 1	5′ GAAGACGACAGGTTCTAACAT 3′
Seq 2	5′ GCTGCAGAGCAAGCTACAGAT 3′
Seq 3	5′ GAAGTCAGACAAGCGGTAGAA 3′

**shRNA design: http://www.sirnawizard.com/siRNA.php.
